# Latent trajectories in autistic individuals: A systematic review

**DOI:** 10.1177/13623613251370818

**Published:** 2025-09-25

**Authors:** Kamil R Hiralal, Gwendolyn C Dieleman, Britt R Kok, Luka D Diederen, Rana P Duman, Manon HJ Hillegers, Sabine E Mous

**Affiliations:** Erasmus Medical Center—Sophia Children’s Hospital, The Netherlands

**Keywords:** autism spectrum disorder, development, heterogeneity, systematic review

## Abstract

Autism is heterogenic in core and co-occurring characteristics. Subtyping autism in a longitudinal manner aids the understanding of autism development throughout life and thus enhances personalized support. In this systematic review, we summarized the literature on latent core autism characteristics trajectories and trajectories of other functional domains in autistic individuals and identified predictors of trajectory assignment. We searched Embase, Medline, PsycINFO, Cochrane Central, Web of Science, and Google Scholar until April 22, 2025. We included longitudinal observational studies that applied statistical subtyping methods on core autism characteristics or other functional domains in autistic individuals. A total of 30 eligible analyses were included. The included analyses investigated core autism characteristics (10), adaptive behavior (10), behavioral problems (7), adverse childhood experiences (1), cognitive development (1), and feeding problems (1). For each domain, we found differing numbers and shapes of trajectories. Cognitive development was predictive of core autism symptom trajectory classifications, where cognitive development was generally lower in more severely affected core autism symptom trajectories. We found mixed results for other predictors. Future studies should focus on understudied outcome domains, such as motor coordination or sleep problems. In addition, more research is needed to understand when and why individuals deviate from their subgroup trajectory.

**Lay abstract:**

Autistic people can have very different characteristics. Investigating groups based on their characteristics over time can improve our understanding of how autistic people develop and why development can differ between people. We reviewed studies that group autistic individuals based on their development of autistic features and other characteristics. We included 30 analyses and summarized their findings. The studies show that there are different ways autistic individuals develop based on core autistic characteristics (social difficulties and focused, intense and repetitive behaviors, interests and activities), as well as for adaptive behavior, behavioral problems, cognitive development, and feeding problems. For core characteristics, lower cognitive abilities seemed to be related to less favorable developmental pathways. This review showed that autistic people may show distinct patterns of development in core characteristics and other domains. We also highlight that some domains of functioning, such as motor coordination and sleeping problems, are not studied in the literature and future studies should focus on these domains as well since these are difficulties that autistic people often face. Identifying distinct developmental patterns in autistic children can help to predict the outcome of autistic people and may aid in offering personalized support.

## Introduction

To meet *Diagnostic and Statistical Manual of Mental Disorders* (5th ed.; DSM-5; American Psychiatric Association, 2013) criteria for autism spectrum disorder (hereafter: autism), individuals must have difficulties with social interaction and communication, along with focused, intense and repetitive behaviors, interests and activities. The severity of difficulties can vary strongly between and within autistic individuals. By statistically computing longitudinal subgroups, we can identify autistic individuals who show similar development of their symptom manifestation and understand which characteristics set these subgroups apart. Ultimately, this may improve outcome predictions, guide personalized support, and enhance our understanding of autism’s etiology.

Many studies have examined cross-sectional autism subgroups using core autism traits and other functional domains. Agelink van Rentergem et al. (2021) found that most subtyping studies identified two to four subgroups. While core traits are studied most frequently, domains like cognition (Ben-Sasson et al., 2008) and adaptive behavior (Nevill et al., 2017) have also been explored. A limitation of cross-sectional analyses is that they do not take into account temporal changes in autism characteristics and the timing of onset and severity of a variety of characteristics that can be expected from a developmental perspective. Although cross-sectional studies can use age as a covariate and use this to model characteristics over time, they fail in dissecting true temporal effects from cohort effects. For example, the development of two individuals that are born in different eras may diverge due to cultural differences.

Georgiades et al. (2017) coined “chronogeneity” to describe how autism symptoms development varies within and between individuals, consisting of intra- and interindividual heterogeneity. Intraindividual heterogeneity is the variability in development between different characteristics *within* an individual over time. That is, symptom A might become more severe over time, while symptom B remains stable. Interindividual heterogeneity refers to the longitudinal variability in symptom severity *between* individuals: Individuals 1 and 2 may show symptom improvements over time, while Individuals 3 and 4 may show symptom worsening. Interindividual heterogeneity can be studied using longitudinal subtyping methods. Due to time and resource demands, fewer studies have investigated longitudinal heterogeneity in autism populations compared with cross-sectional studies.

**Figure 1.**
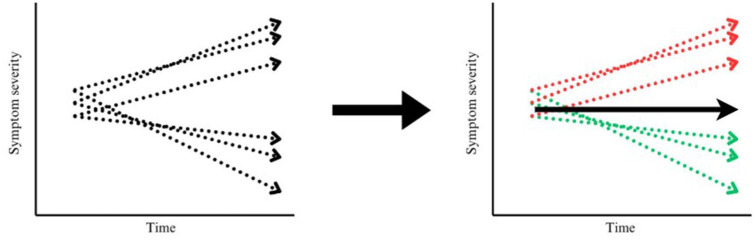
Longitudinal trajectory modeling. After a modeling algorithm, it becomes clear that one group increases in symptom severity and one group decreases in symptom severity, while the overall mean may indicate stable symptom severity.

Longitudinal subgroups can be identified using latent trajectory modeling. This technique aims to group individuals based on shared developmental pathways, where individuals who are classified into the same trajectory group show similar patterns of development (Figure 1). A popular approach to identify latent trajectories is growth mixture modeling, which aims to model a latent categorical variable that determines the trajectory of a variable over time (Ram & Grimm, 2009).

### Co-occurring difficulties in autistic individuals

Next to core autism characteristics, autistic individuals often experience other difficulties, such as cognitive challenges and co-occurring psychopathology (for a review, see Lai et al., 2019). A recent large-scale study suggests that more than 70% of autistic individuals have at least one co-occurring condition, most commonly attention deficit hyperactivity disorder (ADHD; Khachadourian et al., 2023). Understanding the development of both core and co-occurring difficulties is essential for improving outcomes. Moreover, autism stakeholders indicate that better comprehension of autism-related mental health issues should be a research priority (for a review, see Roche et al., 2020).

### Current systematic review

No systematic review has focused on latent symptom trajectories in autistic individuals. However, one systematic review has already investigated the longitudinal heterogeneity of autism characteristics (Pender et al., 2020), including several studies using latent trajectory modeling. Pender et al. (2020) concluded that there is evidence for four distinct trajectories of autistic characteristics. However, the authors only included studies investigating the trajectories of core autism traits. Examining studies which also include other functional domains, next to core autism characteristics, would provide insights into latent trajectories within the range of behavioral features associated with autism.

We aim to systematically review the literature on latent trajectories of core autism characteristics and other functional domains in autistic individuals. Hereby we take into account that autism is not only highly heterogenic in symptom severity, but also in symptom manifestation. From a clinical perspective, it would be beneficial to be able to predict the course of symptom development in autistic individuals based on their trajectory assignment. This can help to target support and optimize outcomes. Therefore, we also aim to identify characteristics that are associated with patterns of symptom change within domains.

## Methods

The results of this systematic review are reported according to the PRISMA 2020 statement (Page et al., 2021). The study protocol was preregistered on PROSPERO (registration number: CRD42023397902).

### Inclusion criteria

We included longitudinal observational studies investigating latent trajectories of core autism characteristics or other functional domains in autistic individuals. Inclusion criteria were the following:

1. The study used a longitudinal mixture model to identify latent trajectories.
2. The study sample consisted of individuals with a DSM (American Psychiatric Association, 2013) classification of autism spectrum disorder, autism, Asperger’s syndrome, and/or PDD-NOS (pervasive developmental disorder–not otherwise specified).

We used the following exclusion criteria:

1. Investigation of trajectories of somatic characteristics (e.g. cardiovascular issues or brain volume).
2. The study was not written in English.
3. The study was not peer-reviewed.

When a study was not eligible for inclusion based on the complete sample (e.g. mixed sample of autism patients and healthy controls) but contained a subgroup analysis that fulfilled our inclusion criteria, we included the subgroup analysis in our study sample.

### Search strategy and selection procedure

The search strategy was developed in consultation with a medical librarian from the Erasmus Medical Center. We conducted our first search on November 3, 2022, and repeated our search on April 22, 2025. The following databases were used: *Medline Ovid*, *Embase*, *Web of Science*, *Cochrane Central Register of Controlled Trials*, and *PsycINFO* (see Supplementary Material for our complete search strategy). Two authors independently performed title and abstract screening on all identified articles. Next, the full texts of all remaining articles were screened by two authors independently. After each step, discrepancies were agreed upon after thorough discussion. Finally, we screened the reference lists of the included articles for relevant studies, but this did not result in additional articles.

### Data collection

The main outcome of interest was the number of identified latent trajectories. We also extracted predictors of trajectory assignment. If a study included analyses of multiple domains (e.g. adaptive behavior and anxiety), we extracted data from all analyses of a study and regarded them as separate in our systematic review. To summarize the identified trajectories, we collected data on the intercepts (e.g. low or high) and the shape of the trajectory (e.g. linear decrease or quadratic increase).

We only included the most recent article in our review if a sample was analyzed in multiple articles using the same outcome measure (e.g. follow-up studies). Data collection was done by two authors independently and any discrepancies were checked and agreed upon.

### Risk of bias assessment

To assess the risk of bias, we used the Guidelines for Reporting on Latent Trajectory Studies (GRoLTS; van de Schoot et al., 2017). The GRoLTS-checklists consist of 21 items which assess whether a study reports adequately on their modeling steps. Studies that had a high risk of bias were excluded from further analyses. Note that the use of the GRoLTS deviates from our preregistration, as we only came across this instrument after preregistering our protocol.

### Data synthesis procedure

In order to compare results between studies, we grouped studies based on their outcome domain. To our knowledge, there is no meta-analytic method to analyze the number of latent trajectories across studies. Therefore, we chose to present a narrative synthesis of our findings.

## Results

### Study characteristics

**Figure 2.**
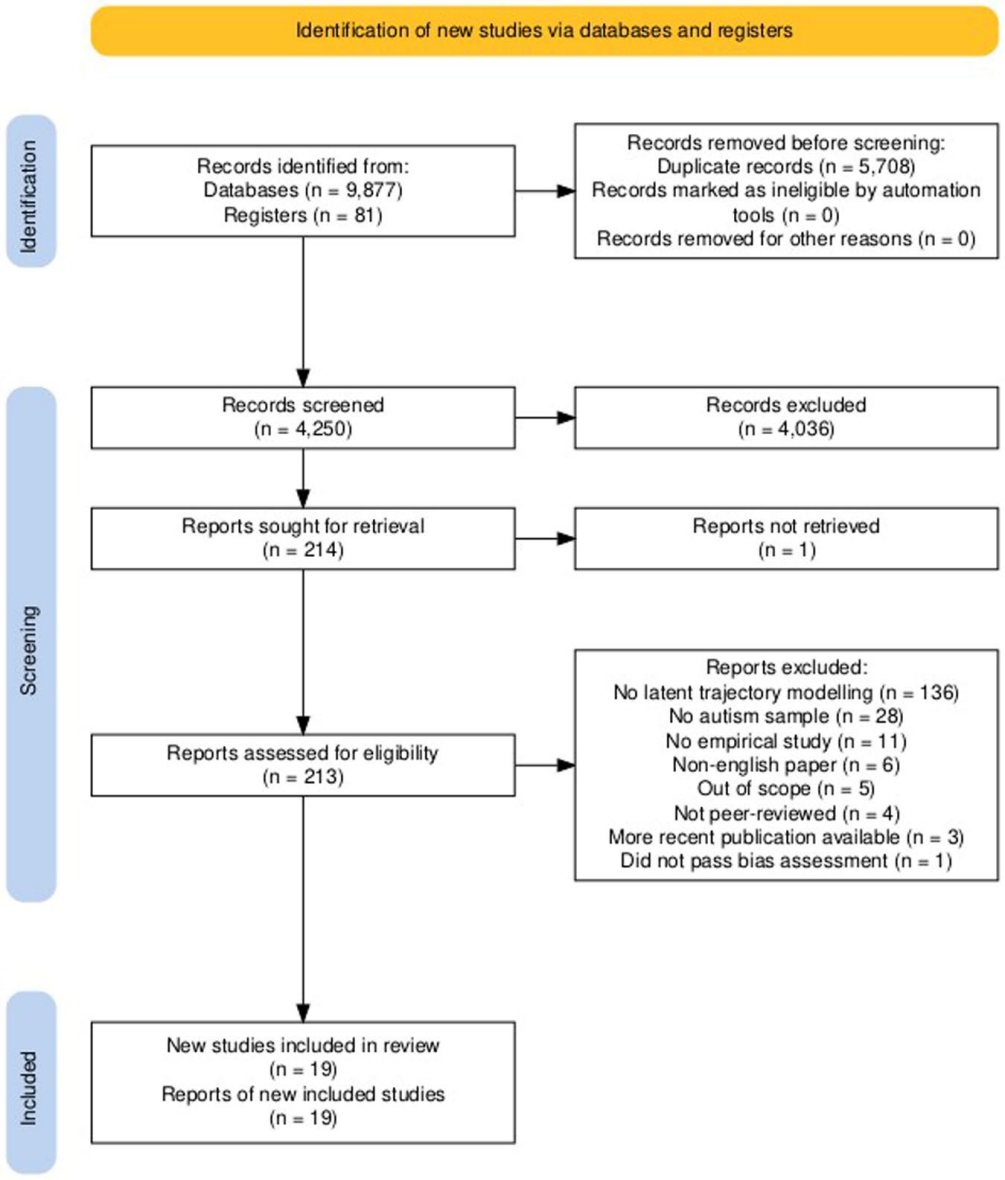
Flow diagram of the screening procedure (Haddaway et al., 2022).

**Figure 3.**
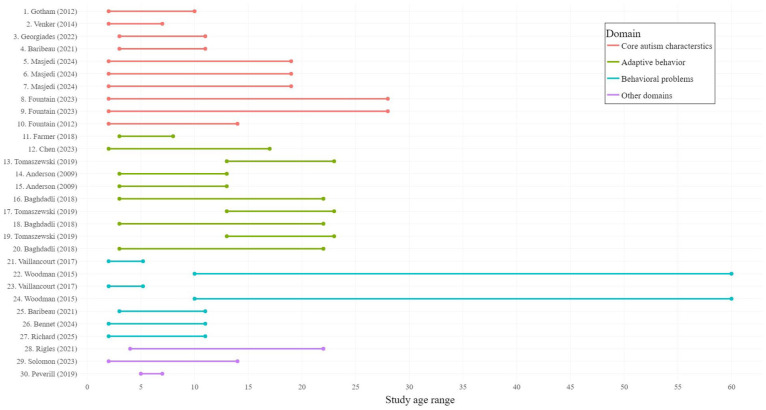
Age ranges for included analyses. Data were extracted from the tables and figures from the included studies and reflect the approximate study period over which latent trajectories were identified.

The search strategy yielded 4250 unique articles. After excluding 4036 articles based on their title and abstract, we evaluated the full text of 214 articles. We identified 23 eligible articles, although one article (Smith et al., 2007) did not pass our risk of bias assessment. Next, we excluded several analyses since more recent analyses of the same outcome variable were available from more recent papers (Baghdadli et al., 2012; Fountain et al., 2012; Richler et al., 2010; Solomon et al., 2018; Szatmari et al., 2015). This resulted in 30 included analyses (Tables 1–4) from 19 different studies (Figure 2) with a median sample size of 244 participants. For some outcome domains, several measures were used across studies and the intercepts and growth patterns should not be directly compared. The majority of the analyses focused on latent symptom trajectories of core autism characteristics (10) and adaptive behavior (10), followed by behavioral problems (7), adverse childhood experiences (1), cognitive functioning (1), and feeding problems (1). The age range for each analysis is depicted in Figure 3.

**Table 1.** Study summaries—core autism characteristics.

Analysis	Cohort	N	Male/female distribution	Instrument	Subscale	Intercept + growth pattern (%)
1. Gotham et al. (2012)	Early diagnosis of ASD	345	81.7/18.3	ADOS	Total CSS	1: high + stable (46.0) 2: moderate + stable (38.8) 3: mild + linear increase (8.8) 4: moderate + linear decrease (6.8)
2. Venker et al. (2014)	Study-specific sample	129	86.8/13.2	ADOS	Total CSS	1: high + stable (36.4) 2: moderate + stable (41.8) 3: mild + linear increase (7.8) 4: moderate + linear decrease (14.0)
3. Georgiades et al. (2022)	Pathways in ASD	187	85.6/14.4	ADOS	All CSS	1: moderate + stable (72.7) 2: moderate + linear decrease (27.3)
4. Baribeau et al. (2021)	Pathways in ASD	409	84.4/15.6	ADI-R	Insistence on sameness	1: high + quadratic increase (6.2) 2: moderate + linear increase (52.0) 3: low + stable (41.7)
5. Masjedi	Early diagnosis of ASD	193	86.5:13.5	ADI-R	Insistence on sameness	1: high + quadratic decrease (18.5) 2: low + cubic increase (81.5)
6. Masjedi	Early diagnosis of ASD	193	86.5:13.5	ADI-R	Repetitive sensorimotor behavior	1: high + quadratic increase (27.3) 2: moderate + linear decrease (44.4) 3: low + linear decrease (28.2)
7. Masjedi	Early diagnosis of ASD	127	86.6:13.4	ADI-R	Verbal repetitive behavior	1: low + quadratic increase (46.5) 2: low + stable (53.5)
8. Fountain et al. (2023)^a^	CDDS	71,222	82.2/17.8	CDER	Communication	1: moderate + increasing s-curve (22.0) 2: moderate + quadratic increase (13.8) 3: low + stronger increasing s-curve (29.9) 4: low + increasing s-curve (12.5) 5: low + quadratic increase (13.2) 6: low + stable (8.6)
9. Fountain et al. (2023)^a^	CDDS	71,184	82.2/17.8	CDER	Socialization	1: moderate + quadratic decrease (5.0) 2: moderate + quadratic decrease (33.7) 3: mild + stable (21.4) 4: low-mild + stronger quadratic increase (10.1) 5: low-mild + quadratic decease (3.2) 6: low-mild + quadratic increase (19.6) 7: low + quadratic increase (7.0)
10. Fountain et al. (2012)^a^	CDDS	6975	82.0/18.0	CDER	Repetitive behavior	1: high + stable (21.4) 2: high + decreasing s-curve (7.1) 3: mild-moderate + linear increase (28.0) 4: mild-moderate + increasing s-curve (8.1) 5: mild + stable (27.6) 6: low + stable (7.8)

*Note.* Trajectories are listed from highest to lowest intercept. ADOS = Autism Diagnostic Observation Schedule; CSS = Calibrate Severity Score; ADI-R = Autism Diagnostic Interview–Revised; CDDS = California Department of Social Services; CDER = Client Development Evaluation Form.

a Higher scores indicate better functioning.

**Table 2.** Study summaries—adaptive behavior.

Analysis	Cohort	N	Male/female distribution	Instrument	Subscale	Intercept + growth pattern (%)
11. Farmer et al. (2018)^a^	Study-specific sample	105	86.6/13.4	VABS	Composite	1: moderate + stable (27.0) 2: low + quadratic decrease (73.0)
12. Chen et al. (2023)^a^	Pathways in ASD	406	84.2/15.8	VABS	Communication, Socialization, Daily living skills	1: moderate + quadratic increase (16.3) 2: mild + quadratic decrease (34.5) 3: low + quadratic decrease (27.8) 4: low + linear decrease (21.4)
13. Tomaszewski et al. (2019)^a^	CSESA	244	84.8/15.2	VABS	Socialization	1: moderate + linear increase (82.0) 2: low + stable (18.0)
14. Anderson et al. (2009)^a,b^	Early diagnosis of ASD	93	Not reported	VABS	Socialization (age equivalent)	1: low + linear increase (20.4) 2: low + stable (89.6)
15. Anderson et al. (2009)^a,b^	Early diagnosis of ASD	51	Not reported	VABS	Socialization (age equivalent)	1: low + linear increase (19.6) 2: low + small linear increase (80.4)
16. Baghdadli et al. (2018)^a^	EpiTED	94	86.0/14.0	VABS	Socialization	1: low + linear increase (21.0) 2: low + stable (79.0)
17. Tomaszewski et al. (2019)^a^	CSESA	244	84.8/15.2	VABS	Daily living skills	1: moderate + linear increase (89.0) 2: low + stable (11.0)
18. Baghdadli et al. (2018)^a^	EpiTED	94	86.0/14.0	VABS	Daily living skills	1: low + linear increase (19.3) 2: low + stable (80.7)
19. Tomaszewski et al. (2019)^a^	CSESA	244	84.8/15.2	VABS	Communication	1: moderate + linear increase (87.0) 2: low + stable (13.0)
20. Baghdadli et al. (2018)^a^	EpiTED	94	86.0/14.0	VABS	Communication	1: low + strong linear increase (21.5) 2: low + stable (78.5)

Trajectories are listed from highest to lowest intercept. CSESA = Center on Secondary Education for Students with Autism Spectrum Disorder; EpiTED = Epidemiological Study of Outcome of Children with Pervasive Developmental Disorders; VABS = Vineland Adaptive Behavior Scales.

a Higher scores indicate better functioning.

b Anderson et al. (2009) conducted two analyses (autism and PDD-NOS) that fulfilled the inclusion criteria.

**Table 3.** Study summaries—behavioral problems.

Analysis	Cohort	N	Male/female distribution	Instrument	Subscale	Intercept + growth pattern (%)
21. Vaillancourt et al. (2017)	Pathways in ASD	105	86.6/13.4	CBCL	Internalizing problems	1: high + stable (23.2) 2: low + linear decrease (76.8)
22. Woodman et al. (2016)	Adults with Autism study	259	74.9/25.1	ABCL	Internalizing problem	1: moderate-high + linear decrease (9.4) 2: moderate + linear decrease (20.3) 3: mild + stable (33.3) 4: low + stable (37.0)
23. Vaillancourt et al. (2017)	Pathways in ASD	244	84.8/15.2	CBCL	Externalizing problems	1: high + stable (13.5) 2: moderate + linear decrease (46.4) 3: low + linear decrease (40.1)
24. Woodman et al. (2016)	Adults with Autism study	259	74.9/25.1	ABCL	Externalizing problems	1: moderate-high + linear decrease (14.4) 2: mild + stable (34.1) 3: low + stable (51.5)
25. Baribeau et al. (2021)	Pathways in ASD	399	Not reported	CBCL	Anxiety	1: high + stable (13.1) 2: moderate + quadratic decrease (16.2) 3: moderate + quadratic increase (19.6) 4: low + cubic increase (51.0)
26. Bennett et al. (2025)	Pathways in ASD	396	84.1/15.9	ABC	Emotional dysregulation	1: high + stable (18.0) 2: moderate + linear decrease (54.0) 3: low + stable (28.0)
27. Richard et al. (2025)	Pathways in ASD	393	84.0/16.0	CBCL	Attention problems	1: high + stable (7.0) 2: moderate + linear decrease (33.0) 3: low + linear decrease (25.0) 4: low + linear increase (19.0) 5: low + stable (15.0)

Trajectories are listed from highest to lowest intercept. ABC = Aberrant Behavior Checklist; ABCL = Adult Behavior Checklist; CBCL = Child Behavior Checklist.

**Table 4.** Study summaries—other domains.

Analysis	Cohort	N	Male/female distribution	Instrument	Subscale	Intercept + growth pattern (%)
**Adverse childhood experiences**						
28. Rigles (2021)	Study-specific sample	902	74/26	Study questionnaire	Adverse childhood experience	1: high + quadratic increase (25.1) 2: moderate + stable (22.3) 3: low + stable (52.5)
**Cognitive functioning**						
29. Solomon et al. (2023)^a^	Autism Phenotype Project	152	82.2/17.8	MSEL/DAS-II	Cognitive development	1: average + quadratic increase (16.0) 2: below average + quadratic increase (39.0) 3: low + stable (45.0)
**Feeding problems**						
30. Peverill et al. (2019)	Pathways in ASD	152	82.2/17.8	BPFAS	Feeding problems	1: very high + stable (8.3) 2: high + linear decrease (26.5) 3: moderate + linear decrease (38.9) 4: mild + stable (26.3)

Trajectories are listed from highest to lowest intercept. BPFAS = Behavioral Pediatrics Feeding Assessment Scale; DAS-II = Differential Ability Scales–II; MSEL = Mullen Scales of Early Learning.

a Higher scores indicate better functioning.

The Early Diagnosis of ASD study has also included participants who never received an ASD diagnosis, but were diagnosed with a nonspectrum developmental delay. Therefore, most of the studies using data from this cohort were excluded from this review (Anderson et al., 2007, 2011; Bal et al., 2015; Christopher et al., 2023; Clarke, McCauley, & Lorde, 2021; Clarke, Sterret, & Lord, 2021, 2023; Kim et al., 2018; Lord et al., 2012; McCauley et al., 2020; Pickles et al., 2014; Richler et al., 2010). The studies that have used only data from autistic participants were included in this study.

### Core autism characteristics

The three analyses that used the Autism Diagnostic Observation Scale (ADOS; Gotham et al., 2006) found the following: Georgiades et al. (2022) identified a severe and stable trajectory and an improving trajectory, while Gotham et al. (2012) and Venker et al. (2014) found a severe and stable, a moderately severe and stable, an improving, and a worsening trajectory. In addition, the majority of the samples (ranging from 73% to 84%) in all these analyses were classified within stable trajectories and only small proportions of the samples showed change in symptom severity.

Four analyses used the Autism Diagnostic Interview–Revised (ADI-R; Lord et al., 1994) to investigate the autism characteristics. Both Baribeau et al. (2021) and Masjedi et al. (2024) found the majority of the sample to show increasing severity (58% and 82%, respectively). Baribeau et al. (2021) found a final stable trajectory, whereas Masjedi et al. (2024) found a decreasing severity trajectory. For the domain of repetitive sensorimotor behaviors, Masjedi et al. (2024) found the majority of the sample to display decreasing trajectories, while the rest of the sample fluctuated over time. Finally, Masjedi et al. (2024) found about half of the sample to show an increase in verbal repetitive behavior, while the other half remained stable over time. In sum, the ADI-R analyses indicate two to three latent trajectories of core autism characteristics. Contrary to the ADOS analyses, the ADI-R findings suggest more change in core autism characteristics over time.

Three analyses used a client development evaluation report, measuring difficulties with communication, social behavior, and repetitive behavior. For the communication and social domains, the analyses revealed improving trajectories and stable trajectories (Fountain et al., 2012, 2023), while there also was a worsening trajectory for the repetitive behavior domain (Fountain et al., 2012). While these results imply multiple trajectories of core autism symptom severity, a client development evaluation report is not intended for diagnostic purposes (Fountain et al., 2012) and the results from these analyses should be interpreted with care.

### Adaptive behavior

Two analyses used the Vineland Adaptive Behavior Scales (VABS; Sparrow et al., 2005) to investigate total adaptive behavior. Chen et al. (2023) and Farmer et al. (2018) found 84% and 73% of the sample in an increasing severity trajectory, respectively. The rest of the trajectories remained stable or improved. Four analyses investigating social adaptive behavior trajectories using the VABS identified a trajectory that improved over time and another that remained (nearly) stable or worsened (Anderson et al., 2009; Baghdadli et al., 2012; Tomaszewski et al., 2019). Three of these analyses found that the smallest trajectory showed the most improvements (20%–21%), while the other analysis found the majority of the sample showing improvements (82%). Next, two studies using the daily living skills subscale found a low and stable daily living skills trajectory and an improving trajectory (Baghdadli et al., 2018; Tomaszewski et al., 2019), however the proportion of participants showing improvements differed strongly (81% vs. 11%, respectively). Two studies used the communication subscale of the VABS and identified a low and stable trajectory and an improving trajectory (Baghdadli et al., 2018; Tomaszewski et al., 2019). While one study showed 13% to have stable communicative adaptive behavior, the other study indicated 79% of the participants with no improvements. In sum, there seem to be at least two trajectories of adaptive behavior. Most analyses found a trajectory showing improvements in adaptive behavior and a trajectory that remains stable over time.

### Behavioral problems

Five analyses investigated trajectories of behavioral problems using the Child Behavior Checklist (CBCL; Achenbach, 1999) and two used the Adult Behavior Checklist (ABCL; Achenbach & Rescorla, 2003). First, Vaillancourt et al. (2017) and Woodman et al. (2016) found stable and decreasing severity trajectories of internalizing problems. Vaillancourt et al. (2017) found 23% of the sample to show stable severity, whereas 70% in Woodman et al. (2016) showed stable severity. Second, Vaillancourt et al. (2017) and Woodman et al. (2016) found stable and decreasing severity trajectories of externalizing problems. In both studies, the majority sample showed low to mild problem behavior that remained stable or declined. Third, Baribeau et al. (2021) identified four latent trajectories of anxiety with 84% of the sample showing a severe and stable or an increasing severity trajectory. Finally, Richard et al. (2025) found five trajectories of attentional problems that showed either stable problems or improvements over time. Next, Bennet et al. (2025) used the Aberrant Behavior Checklist (ABC; Aman et al., 1985) and found three trajectories of emotional dysregulation problems. The majority (82%) of the sample showed improvements over time or already showed a low degree of emotional dysregulation problems.

### Adverse childhood experiences

Rigles (2021) found three trajectories of a number of adverse childhood experiences. About a quarter of the sample showed a high and increasing trajectory, while the other groups remained stable over time.

### Cognitive functioning

One analysis investigated trajectories of cognitive development using the Mullen Scales of Early Learning (MSEL; Mullen, 1995) and the Differential Abilities Scales (DAS; Elliott et al., 2018). Solomon et al. (2023) identified a small trajectory group (16%) that showed strong improvements over time, while the other groups exhibited various degrees of developmental delays.

### Feeding problems

Finally, Peverill et al. (2019) found four latent trajectories of feeding problems using the Behavioral Pediatrics Feeding Assessment Scale (BPFAS; Crist et al., 1994): two improving trajectories (combined 65%), one severe and stable trajectory (8%), and one trajectory with low and stable feeding problems (26%).

### Predictors of trajectory assignment

Most included analyses tested for baseline predictors of trajectory assignment. Supplementary Material S1 presents a complete list of all the tested predictors. Here we report on the predictors that were most frequently tested.

Cognitive functioning was related to trajectory membership in 18 of the 25 analyses (Anderson et al., 2009; Baghdadli et al., 2018; Bennet et al., 2025; Chen et al., 2023; Fountain et al., 2012, 2023; Georgiades et al., 2022; Gotham et al., 2012; Richard et al., 2025; Tomaszewski et al., 2019; Venker et al., 2014). All but one of the analyses found cognition to be higher in the less severely affected trajectories.

Only three of 27 analyses found a significant association between biological sex and trajectory severity (Fountain et al., 2023; Rigles, 2021; Vaillancourt et al., 2017). In addition, the analyses that did find an effect showed mixed results on whether males or females were more severely affected.

Socioeconomic factors appeared to be somewhat related to trajectory assignment. First, nonwestern ethnicity was associated with classification in more severe trajectories in three of 14 of the analyses testing this association (Fountain et al., 2012, 2023), but this effect was only found for the core symptom trajectories. Second, caregiver education was higher in less severe trajectories in six of the 14 analyses testing for this association (Anderson et al., 2009; Bennet et al., 2025; Fountain et al., 2012, 2023; Woodman et al., 2016). Again, this effect was mostly observed for the core symptom trajectories. Third, family income was higher in less affected trajectories in five of nine analyses (Bennet et al., 2025; Peverill et al., 2019; Rigles, 2021; Vaillancourt et al., 2017). Fourth, the house value of individuals in the least affected trajectories was higher in both analyses testing for this effect (Fountain et al., 2023). Finally, school quality was not associated with trajectory assignment in the three analyses testing for this association (Tomaszewski et al., 2019). In sum, there is some evidence that better socioeconomic status is related to being assigned to less severe symptom trajectories, especially with regard to core autism characteristics trajectories.

Autism characteristics severity was associated with trajectory assignment in 10 of 20 analyses. More pronounced autism characteristics at baseline were related to the more severely affected trajectories of adaptive behavior, anxiety, and cognitive development (Baghdadli et al., 2018; Baribeau et al., 2021; Masjedi et al., 2024; Rigles, 2021; Solomon et al., 2023; Tomaszewski et al., 2019).

Finally, there was some evidence for an association between language development and trajectory severity. In six of 11 studies, language development was better in the least affected trajectories (Anderson et al., 2009; Baghdadli et al., 2018; Georgiades et al., 2022).

## Discussion

This is the first systematic review that focused on studies investigating a range of behavioral features associated with autism, instead of only looking at core autism characteristics. We found evidence for the existence of distinct symptom trajectories across domains. Regarding core autism characteristics severity, the analyses using validated measures suggest the existence of two to five latent trajectories of severity. The majority of individuals show stable characteristics regarding overall autism severity, as measured with the ADOS. However, patterns of focused, intense, and repetitive behaviors, interests, and activities display that most individuals show increasing severity.

In addition to previous work, we showed that the majority of the analyses investigating adaptive behavior identified two trajectories, providing evidence for a trajectory that shows low and stable adaptive behavior and an improving trajectory. There seemed to be no clear patterns in the distributions of trajectory classifications, even within the same subdomains of adaptive behavior. Regarding behavioral problems, most autistic individuals seem to improve or remain stable. However, for anxiety and attentional problems, there were trajectories of increasing severity. Again, there was quite some variance in the amount of individuals who improved compared with those who did not. Although there is evidence for distinct latent trajectories of adverse childhood events, cognitive development, and feeding problems, there was only one study for each domain and these results should be interpreted with care.

Our review included two studies that were also included in Pender et al. (2020), and our findings—showing that the development of autistic individuals can be subdivided into distinct subtrajectories—are in line with Pender et al. (2020). Finally, we identified baseline characteristics that are associated with trajectory assignment, with cognitive development being the most clear predictor of symptom trajectories.

The variability in the number of and distribution across identified trajectories may be explained by several reasons. First, the sampling timing and age range varied across studies, while it is known that these can both influence the number of trajectories (Eggleston et al., 2004). Indeed, the age ranges in the included studies differ strongly. As individuals get older, a variety of environmental factors may influence development, which may give rise to differing number of trajectories at different stages of life. Regarding the studies included in this review, it is clear that the development of adults in mid-to-late adulthood is severely understudied. Second, the included studies use cohorts from different countries and cultural differences can influence the development of autistic individuals (Bernier et al., 2010). Third, the use of different measures for the same outcome domain hinders the comparability between studies.

Our research indicates that distinct trajectories of autistic individuals exist. By identifying factors associated with these trajectories, we can enhance our knowledge of the factors that drive these differences between autistic individuals. Our findings support the need to view autism as an umbrella condition (Geschwind & Levitt, 2007; Happé et al., 2006), where individuals with the same diagnosis can differ strongly in symptom severity and manifestation over time.

### Predictors of trajectory assignment

Baseline cognition levels are generally lower in trajectories that show worsening in core autism characteristics and adaptive behavior. This clustering of core autism characteristics, adaptive behavior trajectories and cognition may inform us on the biological underpinnings of how a range of behavioral features that are associated with autism are interrelated. For example, core characteristics, cognitive development, and adaptive behavior may have directional and temporal relationships, with genetic factors also influencing this complex interplay (Mollon et al., 2021; Tucker-Drob et al., 2013). It is important to note that the cognitive level of an autistic individual can strongly influence whether they participate in research or not (Russel et al., 2019). Therefore, latent autism trajectories of the studies in the current systematic review may not be representative of the full autistic spectrum.

Although there is a large difference in the prevalence of autism classifications between males and females (Loomes et al., 2017; Narzisi et al., 2018), we found no evidence that biological sex is related to the severity of symptom trajectories. These findings are consistent with studies investigating sex differences in autism severity on a cross-sectional level (Dellapiazza et al., 2022; Frazier et al., 2014). There is ongoing debate on how to accurately measure autism characteristics in females (Gould, 2017; Kentrou et al., 2024). Due to the different manifestations of autism between males and females, there is a gender bias in autism classification where females are less likely to be diagnosed as compared with males (Loomes et al., 2017). Therefore, it is likely that females with a less-pronounced phenotype are underrepresented in clinical samples, which in turn may bias our views on how sex is related to longitudinal symptom trajectories in autistic individuals.

Individuals from parents with lower socioeconomic status may have an increased risk of being underdiagnosed with autism due to lower access to mental healthcare systems (Kelly et al., 2019). For example, ethnic minorities have lower access to mental healthcare, leading to inequality in diagnoses and treatment (Lu et al., 2021). Recent studies show that this inequality is present for access to autism services (Lindsay et al., 2024; Liu et al., 2023). The effects of socioeconomic status on mental healthcare may lead to an underrepresentation of individuals with lower socioeconomic status in clinical samples and study populations, especially individuals with lower support needs who may be less likely to be diagnosed with autism. Consequently, this may lead to an overestimation of the effect of socioeconomic indicators found in research. Therefore, the socioeconomic status of a study sample is an important factor for the evaluation of a possible selection bias.

### Strengths and limitations

This systematic review is the first to include studies investigating a range of behavioral features associated with autism. Hereby we acknowledge the problems that autistic individuals may experience in other behavioral domains and increase our knowledge of this complex phenotype. Furthermore, by only including clinical samples in our review, our results may be more easily translated to clinical practice, as when we would have included general population studies.

Our review also has limitations. We used the GRoLTS-checklist (van de Schoot et al., 2017) to assess the risk of bias. However, this is not an agreed upon tool to assess the risk of bias, such as the ROBINS-I (Sterne et al., 2016) or PROBAST (Wolff et al., 2019). To our knowledge, there is no established standard for assessing the risk of bias in longitudinal subtyping studies. Furthermore, multiple studies in our review are based on the same cohort, which may have introduced bias in our results. Finally, autistic individuals above the age of 30 are underrepresented in longitudinal trajectory studies and the results can therefore not be generalized to this age group.

### Future directions

Most of the studies in our review investigated trajectories of core autism characteristics and adaptive behavior. In line with stakeholder views on what should be research priorities (Roche et al., 2020), future studies should investigate additional domains that are commonly affected in autistic individuals, such as depressive symptoms (Hollocks et al., 2019), motor coordination (Fournier et al., 2010), or sleeping problems (Devnani & Hegde, 2015). Next, replication studies using the same age range and outcome measures are warranted to increase comparability between studies.

The derivation of symptom trajectories is specific on study samples and individuals may deviate from their assigned trajectory at any given time point (Georgiades et al., 2017). Future studies could adopt the modeling approach of Manrique-Vallier (2014), as proposed by Georgiades et al. (2017), which relaxes the assumption that an individual can only follow one trajectory. This approach allows individuals to switch between trajectories and can help identify when and why individuals deviate from their expected development. This may increase our ability to make personalized predictions of patient outcomes.

Finally, it would be interesting to see which latent trajectories emerge in broader samples, including a range of developmental conditions, as many of the behaviors that are frequently observed in autism are also common in other conditions (Joon et al., 2021).

## Conclusion

There are distinct patterns of life course symptom development across domains in autistic individuals. Specifically for core autism characteristics, individuals may show a similar pattern of symptom development (e.g. stability of overall autism severity), but subgroups can deviate from this expected trajectory. These deviations can be predicted early on, as they may be driven by risk and resilience factors such as cognitive development or socioeconomic status. More research is needed to detect clear patterns across other functional domains. Ultimately, this may contribute to personalized support services.

Autism heterogeneity poses well-recognized challenges for applying research to clinical practice (Lombardo et al., 2019). Strong phenotypic differences between autistic individuals can make study findings sample-specific and hard to replicate. Clinically, it is important to be aware that group-level results might not be reflective for the entire population due to the existence of latent trajectories of autistic individuals. Since trajectory classes can diverge in symptom severity over time, group-level differences may be obscured due to measurement timing. Due to the strongly heterogenic nature of autism, autistic individuals should be regularly assessed on a broad behavioral phenotype in order to accurately track their development and aid prognosis, treatment, and support.

## Supplemental Material

sj-docx-1-aut-10.1177_13623613251370818 – Supplemental material for Latent trajectories in autistic individuals: A systematic review Supplemental material, sj-docx-1-aut-10.1177_13623613251370818 for Latent trajectories in autistic individuals: A systematic review by Kamil R Hiralal, Gwendolyn C Dieleman, Britt R Kok, Luka D Diederen, Rana P Duman, Manon HJ Hillegers and Sabine E Mous in Autism
