# Supplementary material for: Latent trajectories in autistic individuals: A systematic review
Source: Autism. 2025 Sep 25;30(2):285–99. doi: 10.1177/13623613251370818 (PMC12804397; doi:10.1177/13623613251370818)
Supplement: sj-docx-1-aut-10.1177_13623613251370818 – Supplemental material for Latent trajectories in autistic individuals: A systematic review [file sj-docx-1-aut-10.1177_13623613251370818.docx]

Supplementary materials

Latent trajectories in autistic individuals: a systematic review

**Table of content**

1. **Prisma checklist**
2. **Full search strategy**
3. **Supplementary table**
4. **Risk of bias assessment**

**1. PRISMA checklist**

| **Section and Topic** | **Item #** | **Checklist item** | **Location where item is reported** |
| --- | --- | --- | --- |
|  |  |  |  |
| **TITLE** | | |  |
| Title | 1 | Identify the report as a systematic review. | Title |
| **ABSTRACT** | | |  |
| Abstract | 2 | See the PRISMA 2020 for Abstracts checklist. |  |
| **INTRODUCTION** | | |  |
| Rationale | 3 | Describe the rationale for the review in the context of existing knowledge. | Introduction - Current systematic review |
| Objectives | 4 | Provide an explicit statement of the objective(s) or question(s) the review addresses. | Introduction - Current systematic review |
| **METHODS** | | |  |
| Eligibility criteria | 5 | Specify the inclusion and exclusion criteria for the review and how studies were grouped for the syntheses. | Methods - Inclusion criteria |
| Information sources | 6 | Specify all databases, registers, websites, organisations, reference lists and other sources searched or consulted to identify studies. Specify the date when each source was last searched or consulted. | Methods – Search strategy and selection procedure |
| Search strategy | 7 | Present the full search strategies for all databases, registers and websites, including any filters and limits used. | Supplementary materials |
| Selection process | 8 | Specify the methods used to decide whether a study met the inclusion criteria of the review, including how many reviewers screened each record and each report retrieved, whether they worked independently, and if applicable, details of automation tools used in the process. | Methods – Search strategy and selection procedure |
| Data collection process | 9 | Specify the methods used to collect data from reports, including how many reviewers collected data from each report, whether they worked independently, any processes for obtaining or confirming data from study investigators, and if applicable, details of automation tools used in the process. | Methods – Data collection |
| Data items | 10a | List and define all outcomes for which data were sought. Specify whether all results that were compatible with each outcome domain in each study were sought (e.g. for all measures, time points, analyses), and if not, the methods used to decide which results to collect. | Methods – Data collection |
|  | 10b | List and define all other variables for which data were sought (e.g. participant and intervention characteristics, funding sources). Describe any assumptions made about any missing or unclear information. | Methods – Data collection |
| Study risk of bias assessment | 11 | Specify the methods used to assess risk of bias in the included studies, including details of the tool(s) used, how many reviewers assessed each study and whether they worked independently, and if applicable, details of automation tools used in the process. | Methods – Risk of bias assessment |
| Effect measures | 12 | Specify for each outcome the effect measure(s) (e.g. risk ratio, mean difference) used in the synthesis or presentation of results. | Methods – Data synthesis |
| Synthesis methods | 13a | Describe the processes used to decide which studies were eligible for each synthesis (e.g. tabulating the study intervention characteristics and comparing against the planned groups for each synthesis (item #5)). | Methods – Search strategy and selection procedure |
|  | 13b | Describe any methods required to prepare the data for presentation or synthesis, such as handling of missing summary statistics, or data conversions. | Methods – Data synthesis |
|  | 13c | Describe any methods used to tabulate or visually display results of individual studies and syntheses. | Methods – Data synthesis |
|  | 13d | Describe any methods used to synthesize results and provide a rationale for the choice(s). If meta-analysis was performed, describe the model(s), method(s) to identify the presence and extent of statistical heterogeneity, and software package(s) used. | Methods – Data synthesis |
|  | 13e | Describe any methods used to explore possible causes of heterogeneity among study results (e.g. subgroup analysis, meta-regression). | Not applicable |
|  | 13f | Describe any sensitivity analyses conducted to assess robustness of the synthesized results. | Not applicable |
| Reporting bias assessment | 14 | Describe any methods used to assess risk of bias due to missing results in a synthesis (arising from reporting biases). | Not applicable |
| Certainty assessment | 15 | Describe any methods used to assess certainty (or confidence) in the body of evidence for an outcome. | Not applicable |
| **RESULTS** | | |  |
| Study selection | 16a | Describe the results of the search and selection process, from the number of records identified in the search to the number of studies included in the review, ideally using a flow diagram. | Results – study characteristics |
|  | 16b | Cite studies that might appear to meet the inclusion criteria, but which were excluded, and explain why they were excluded. | Results – study characteristics |
| Study characteristics | 17 | Cite each included study and present its characteristics. | Results - Table 1 |
| Risk of bias in studies | 18 | Present assessments of risk of bias for each included study. | Supplementary materials |
| Results of individual studies | 19 | For all outcomes, present, for each study: (a) summary statistics for each group (where appropriate) and (b) an effect estimate and its precision (e.g. confidence/credible interval), ideally using structured tables or plots. | Results |
| Results of syntheses | 20a | For each synthesis, briefly summarise the characteristics and risk of bias among contributing studies. | Not applicable |
|  | 20b | Present results of all statistical syntheses conducted. If meta-analysis was done, present for each the summary estimate and its precision (e.g. confidence/credible interval) and measures of statistical heterogeneity. If comparing groups, describe the direction of the effect. | Not applicable |
|  | 20c | Present results of all investigations of possible causes of heterogeneity among study results. | Not applicable |
|  | 20d | Present results of all sensitivity analyses conducted to assess the robustness of the synthesized results. | Not applicable |
| Reporting biases | 21 | Present assessments of risk of bias due to missing results (arising from reporting biases) for each synthesis assessed. | Not applicable |
| Certainty of evidence | 22 | Present assessments of certainty (or confidence) in the body of evidence for each outcome assessed. | Not applicable |
| **DISCUSSION** | | |  |
| Discussion | 23a | Provide a general interpretation of the results in the context of other evidence. | Discussion |
|  | 23b | Discuss any limitations of the evidence included in the review. | Discussion – strengths and limitations |
|  | 23c | Discuss any limitations of the review processes used. | Discussion – strengths and limitations |
|  | 23d | Discuss implications of the results for practice, policy, and future research. | Discussion - Conclusion |
| **OTHER INFORMATION** | | |  |
| Registration and protocol | 24a | Provide registration information for the review, including register name and registration number, or state that the review was not registered. | Methods |
|  | 24b | Indicate where the review protocol can be accessed, or state that a protocol was not prepared. | Methods |
|  | 24c | Describe and explain any amendments to information provided at registration or in the protocol. | Methods |
| Support | 25 | Describe sources of financial or non-financial support for the review, and the role of the funders or sponsors in the review. | Author statements |
| Competing interests | 26 | Declare any competing interests of review authors. | Author statements |
| Availability of data, code and other materials | 27 | Report which of the following are publicly available and where they can be found: template data collection forms; data extracted from included studies; data used for all analyses; analytic code; any other materials used in the review. | Not applicable |

*From:*  Page MJ, McKenzie JE, Bossuyt PM, Boutron I, Hoffmann TC, Mulrow CD, et al. The PRISMA 2020 statement: an updated guideline for reporting systematic reviews. BMJ 2021;372:n71. doi: 10.1136/bmj.n71. This work is licensed under CC BY 4.0. To view a copy of this license, visit <https://creativecommons.org/licenses/by/4.0/>

### **2. Full search strategy**

### Embase

('autism'/exp OR 'autism assessment'/exp OR (autis* OR Asperger* OR Rett-syndrome* OR (pervasiveNEAR/3 development*-disorder*) OR childhood-disintegrative-disorder*):ab,ti,kw) AND ('latent structure analysis'/exp OR 'cluster analysis'/exp OR 'illness trajectory'/exp OR (((laten* OR cluster*)NEAR/6 (analys*)) OR ((laten*) NEAR/3 (profile* OR longitudinal*)) OR trajector*):ab,ti,kw) NOT[conference abstract]/lim NOT ((animal/exp OR animal*:de OR nonhuman/de) NOT ('human'/exp))

### Medline Ovid

(exp Child Development Disorders, Pervasive/ OR Rett Syndrome/ OR (autis* OR Asperger* OR Rettsyndrome*OR (pervasive ADJ3 development*-disorder*) OR childhood-disintegrativedisorder*).

ab,ti,kf.) AND (Latent Class Analysis/ OR Cluster Analysis/ OR (((laten* OR cluster*) ADJ6 (analys*)) OR ((laten*) ADJ3 (profile* OR longitudinal*)) OR trajector*).ab,ti,kf.) NOT (news OR congres* OR abstract* OR book* OR chapter* OR dissertation abstract*).pt. NOT (exp animals/ NOT humans/)

### PsycINFO Ovid

(exp Autism Spectrum Disorders/ OR Rett Syndrome/ OR (autis* OR Asperger* OR Rett-syndrome* OR (pervasive ADJ3 development*-disorder*) OR childhood-disintegrative-disorder*).ab,ti.) AND (Latent Class Analysis/ OR Latent Profile Analysis/ OR Cluster Analysis/ OR (((laten* OR cluster*) ADJ6 (analys*)) OR ((laten*) ADJ3 (profile* OR longitudinal*)) OR trajector*).ab,ti.) NOT (news OR congres* OR abstract* OR book* OR chapter* OR dissertation abstract*).pt. NOT ((animal.po. OR exp animals/) NOT human.po.)

### Cochrane Central

((autis* OR Asperger* OR Rett NEXT syndrome* OR (pervasive NEAR/3 development* NEXT disorder*) OR childhood NEXT disintegrative NEXT disorder*):ab,ti,kw) AND ((((laten* OR cluster*) NEAR/6 (analys*)) OR ((laten*) NEAR/3 (profile* OR longitudinal*)) OR trajector*):ab,ti,kw) NOT "conference abstract":pt

### Web of Science

TS=(((autis* OR Asperger* OR Rett-syndrome* OR (pervasive NEAR/2 development*-disorder*) OR

childhood-disintegrative-disorder*)) AND ((((laten* OR cluster*) NEAR/5 (analys*)) OR ((laten*) NEAR/2 (profile* OR longitudinal*)) OR trajector*)) NOT ((animal* OR rat OR rats OR mouse OR mice OR murine OR dog OR dogs OR canine OR cat OR cats OR feline OR rabbit OR cow OR cows OR bovine OR rodent* OR sheep OR ovine OR pig OR swine OR porcine OR veterinar* OR chick* OR zebrafish* OR baboon* OR nonhuman* OR primate* OR cattle* OR goose OR geese OR duck OR macaque* OR avian* OR bird* OR fish*) NOT (human* OR patient* OR women OR woman OR men OR man))) NOT DT=(Meeting Abstract OR Meeting Summary)

### Google Scholar

**autism|autistic|Asperger|'Rett syndrome'|'pervasive development|developmental disorder'|'childhood disintegrative disorder' 'latent|cluster analysis'|'latent profile|profiles|longitudinal'|trajectories**

**3. Supplementary table**

| Table S1. Summary table of baseline predictors of group membership | |  | |  |
| --- | --- | --- | --- | --- |
| Predictor | Analysis numbers | | General direction of effect | |
| Cognitive development | **1^1^, 2, 3,** 4, **5**, 6, 7, **8, 9, 10, 12, 13, 14, 15, 16, 17, 18, 19, 20,** 21, 23, 25, **26, 27,** 30 | | Higher cognition scores in the least affected trajectory groups. | |
| Biological sex | 1, 2, 3, 4, 5, 6, 7, **8, 9,** 10, 12, 13, 14, 15, 16, 17, 18, 19, 20, **21,** 22, 23, 24, 25, 26, **28,** 30 | | No strong evidence of association and directions of effect in significant studies are mixed. | |
| *Socio-economic status* |  | |  | |
| Ethnicity | 1, 2, 5, 6, 7, **8, 9, 10,** 12, 13, 14, 15, 17, 19 | | Non-western ethnicity is associated to more severely affected trajectories in about a quarter | |
| Caregiver education | 2, **8, 9, 10,** 12, 13, 14, **15,** 17, 19, 22, **24, 26** | | Higher caregiver education in least affected trajectories in under half of the analyses. | |
| Family income | 3, 4, 12, **21, 23,** 25, **26, 28, 30** | | Higher family income in least affected trajectories in over half of the analyses | |
| House value | **8, 9** | | Higher house value in least affected trajectories in over half of the analyses | |
| School quality | 13, 17, 19 | | No association. | |
| Socio-economic risk | **27** | |  | |
| Autism symptom severity | **5,** 6, 7, 12, **13, 16, 17, 18, 19, 20,** 21, 22, 23, 24, **25,** 26, 27, **28, 29,** 30 | | Higher autism symptom in severity in most severely affected trajectories in about half of the analyses. | |
| Language development | 2, **3,** 4, **14^2^**, **15^3^**, **16, 18, 20,** 25, 26 | | Better language development in least affected trajectories in half of the analyses | |
| Age at diagnosis | **8, 9, 10,** 12, **28,** 30 | | Mixed effect | |
| Age at inclusion | 2, 3, **4, 13, 17, 19,** 22, 24, 25, 26 | | Lower in better outcome trajectories in half of the analyses | |
| Received intervention | **2, 14,** 15, 16, 18, 20, 29 | | No clear association | |
| Adaptive behavior | **3, 4,** 22, 24, 25, **29** | | Better adaptive behavior in better outcome trajectories | |
| Parental mental health | **4, 25, 26, 28** | | Higher in more severely affected trajectories | |
| Caregiver’s age at child birth | 10, **27** | | No clear association | |
| Low birth weight | 10 | | No association | |
| Maternal warmth | **22,** 24 | | Higher in less severely affected trajectories | |
| Maternal criticism | **22, 24** | | Higher in more severely affected trajectories | |
| Anxiety | **4** | | Higher in more severely affected trajectories | |
| Externalizing behavior | 29 | | No association | |
| Internalizing behavior | 29 | | No association | |
| *Note.* All analyses that tested for an association are reported. See table 1 for corresponding analysis numbers. Bold numbers indicate that a predictor was significantly associated to group membership. ^1^ only verbal cognition, ^2^ only expressive language, ^3^ only receptive language, ^5^ Only mentored, parent-implemented structured teaching. | | | | |

**3. Risk of bias assessment**

| **Study** | **GRoLTS points** | **Quality (Good/Fair/Poor)** | **Include (Yes/No)** |
| --- | --- | --- | --- |
| Anderson (2009) | 9 | Fair | Yes |
| Baghdadli (2018) | 9 | Fair | Yes |
| Baribeau (2021) | 13 | Good | Yes |
| Bennet (2024) | 15 | Good | Yes |
| Chen (2023) | 11 | Good | Yes |
| Farmer (2018) | 14 | Good | Yes |
| Fountain (2012) | 9 | Fair | Yes |
| Fountain (2023) | 6 | Fair | Yes |
| Georgiades (2022) | 9 | Fair | Yes |
| Gotham (2012) | 10 | Good | Yes |
| Masjedi (2024) | 9 | Fair | Yes |
| Peverill (2019) | 13 | Good | Yes |
| Richard (2025) | 12 | Good | Yes |
| Rigles (2021) | 6 | Fair | Yes |
| Smith (2007) | 5 | Poor | No |
| Solomon (2023) | 10 | Good | Yes |
| Tomaszewski (2019) | 11 | Good | Yes |
| Vaillancourt (2017) | 10 | Good | Yes |
| Venker (2014) | 10 | Good | Yes |
| Woodman (2016) | 6 | Fair | Yes |
